# Intraocular pressure during handgrip exercise: The effect of posture and hypercapnia in young males

**DOI:** 10.14814/phy2.15035

**Published:** 2021-10-19

**Authors:** Tinkara Mlinar, Polona Jaki Mekjavic, Joshua T. Royal, Tamara Valencic, Igor B. Mekjavic

**Affiliations:** ^1^ Department of Automation, Biocybernetics and Robotics Jozef Stefan Institute Ljubljana Slovenia; ^2^ Jozef Stefan International Postgraduate School Ljubljana Slovenia; ^3^ Eye Hospital University Medical Centre Ljubljana Slovenia; ^4^ Faculty of Medicine University of Ljubljana Ljubljana Slovenia; ^5^ Department of Biomedical Physiology and Kinesiology Simon Fraser University Burnaby British Columbia Canada

**Keywords:** head‐down tilt, intraocular pressure, spaceflight‐associated neuro‐ocular syndrome, visual impairment

## Abstract

**Purpose:**

As part of our investigations of intraocular pressure (IOP) as a potential contributing factor to the spaceflight‐associated neuro‐ocular syndrome using the 6° head‐down tilt (6°HDT) bed rest experimental model, we compared the effect of rest and isometric exercise in prone and supine 6°HDT positions on IOP with that observed in the seated position.

**Methods:**

Ten male volunteers (age = 22.5 ± 3.1 yrs) participated in six interventions. All trials comprised a 10‐min rest period, a 3‐min isometric handgrip exercise at 30% of participant's maximum, and a 10‐min recovery period. The trials were conducted under normocapnic (NCAP) or hypercapnic (F_I_CO_2_ = 0.01; HCAP) conditions, the latter mimicking the ambient conditions on the International Space Station. IOP, systolic and diastolic pressures, and heart rate (HR) were measured during the trials.

**Results:**

Isometric exercise‐induced elevations in HR and mean arterial blood pressure. IOP in the prone 6°HDT position was significantly higher (*p* < 0.001) compared to IOP in supine 6°HDT position and seated trials at all time points. IOP increased with exercise only in a seated HCAP trial (*p* = 0.042). No difference was observed between trials in NCAP and HCAP. IOP in the prone 6°HDT position was constantly elevated above 21 mmHg, the lower limit for clinical ocular hypertension.

**Conclusions:**

IOP in the prone 6°HDT position was similar to IOP reported in astronauts upon entering microgravity, potentially indicating that prone, rather than supine 6°HDT position might be a more suitable experimental analog for investigating the acute ocular changes that occur in microgravity.

## Graphical abstract

Intraocular pressure(IOP) in the prone 6 head‐down tilt (HDT) position was similar to IOP reported in astronauts upon entering microgravity, potentially indicating that prone, rather than supine 6°HDT position might be a more suitable experimental analog for investigating the acute ocular changes that occur in microgravity.

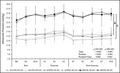

## 1 INTRODUCTION

As with all environmental stressors, the human body also adapts to the microgravity environment, as experienced during spaceflight. The time course of adaptation to microgravity is specific for each physiological system, and the adaptations are typically reversible upon return to Earth's gravity (Nicogossian & Parker Jr, 1982). An exception would appear to be the hypermetropia experienced by astronauts during long‐term missions on the International Space Station (ISS), concomitant with morphological changes: unilateral and bilateral optic disk edema, globe flattening, choroidal and retinal folds, and cotton wool spots (Mader et al., 2011, 2013; Marshall‐Bowman et al., 2013; Nelson et al., 2014). Visual impairment associated with prolonged spaceflight was originally thought to be a consequence of the microgravity‐induced cephalad fluid displacement resulting in increased intracranial pressure (ICP), thus the malady was termed visual impairment/intracranial pressure (VIIP) syndrome (Alexander et al., 2012). Since then, it has been demonstrated that ICP in microgravity is not elevated pathologically (Lawley et al., 2017). Consequently, due to the, as yet, unresolved etiology of this syndrome, VIIP has been redefined as spaceflight‐associated neuro‐ocular syndrome (SANS). Other factors that have been implicated to contribute to the development of SANS include elevated intraocular pressure (IOP), elevated orbital and ventricular cerebrospinal fluid volumes, radiation exposure retinopathy, daily resistive exercise, high sodium diet, and hypercapnia (Alperin & Bagci, 2018; Mader et al., 2011, 2013; Marshall‐Bowman et al., 2013).

While IOP has been reported to increase at the onset of exposure to microgravity (Draeger et al., 1993, 1997), it is doubtful whether the observed IOP of 25 mmHg would instigate the morphological changes observed during longer missions. However, the astronauts’ daily exercise regimen to combat microgravity‐induced sarcopenia may cause further substantial and deleterious increases in IOP. Namely, on Earth, resistive exercise and hypercapnia have both been confirmed to elevate IOP, either independently (Awad et al., 2009; Bakke et al., 2009; Dickerman et al., 1999; Hvidberg et al., 1981; Laurie et al., 2017; Vieira et al., 2006) or synergistically (Kiss et al., 2001; Mekjavic et al., 2020). The elevations in IOP during resistive exercise observed on Earth might be greater when performed in the absence of gravity (in Space), and further exacerbated by the hypercapnic environment experienced on the ISS (Law et al., 2014; Taylor et al., 2013). On Earth, the CO_2_ concentration in ambient air is approximately 0.03%, whereas on the ISS, this number is nominally from 10 to more than 23 times higher (0.3–0.7%) (Alexander et al., 2012). Since 2008, spacecraft maximum allowable concentrations (SMACs) for CO_2_ are defined as 2.0% for 1 h, 1.3% for 24 h, 0.7% for 7–180 days, and 0.5% for 1000 days (James, 2008).

A major problem with studying SANS in ground‐based studies is that current analogs of the effects of microgravity on physiological systems are not appropriate. The experimental bed rest model, in which the subjects are supine in the horizontal or 6° head‐down tilt (HDT) position for different durations, is acceptable for studying the adaptation of most physiological systems to inactivity and unloading of the weight bearing limbs, it has not, however, yielded ophthalmological results similar to those observed on the ISS.

Compared to IOP in a seated position, acute exposure to microgravity (Draeger et al., 1993, 1997) or a supine horizontal or HDT position (Anderson et al., 2016; Carlson et al., 1987; Eklund et al., 2016; Frey et al., 1993; Lam & Douthwaite, 1997; Macias et al., 2015; Mader et al., 1990; Marshall‐Goebel, Mulder, Bershad, et al., 2017; Ozcan et al., 2004; Shinojima et al., 2012) results in increased IOP. This increase is even more pronounced in a prone position (Anderson et al., 2016, 2017; Lam & Douthwaite, 1997; Mekjavic et al., 2020; Ozcan et al., 2004). Questions regarding the suitability of supine or prone HDT positions as analogs for studying effects of microgravity on the ocular system have been raised, particularly given the IOP responses during parabolic flights, which were observed to be in the range between the values occurring in the prone and supine positions (Anderson et al., 2016).

Astronauts are exposed to many external (i.e., ambient conditions) and internal (i.e., microgravity‐induced adjustments in organ systems) factors associated with living on the ISS. Some of these may contribute to the development of SANS. One of the factors that was identified as potentially playing a role in the development of SANS, is elevated IOP. As a prelude to a study investigating the effects of exercise of a duration similar to that conducted on a near‐daily basis by the astronauts on the ISS, we sought to identify the posture that would best mimic the elevations observed in microgravity, and to assess the effect of a short acute bout of resistance exercise on IOP. Specifically, this study assesses the effect of posture (i.e., sitting, supine 6°HDT, and prone 6°HDT) on IOP at rest and during static handgrip exercise at 30% of the participant's maximum strength. Submaximal isometric handgrip exercise was chosen because it has previously been shown to increase both IOP and mean arterial pressure (Bakke et al., 2009; Mekjavic et al., 2020), and enables easier IOP measurements. Due to the reported substantial effect of CO_2_ on IOP in older males (Mekjavic et al., 2020), and the prevalence of hypercapnia on the ISS (Law et al., 2014), we examined the IOP responses also during hypercapnic exercise in a younger male population. The novel approach of assessing IOP while combining and manipulating all three aforementioned factors (i.e., posture, exercise, and hypercapnia) as in this study, has not yet been reported in the existing literature. This study tested the following hypotheses: (i) the resistive exercise‐induced increase in IOP will be greater in the prone compared to the supine 6°HDT position and (ii) resistive exercise‐induced increases in IOP will be exacerbated by hypercapnia.

## 2 METHODS

### 2.1 Participants

Ten healthy, non‐smoking young male participants gave their written informed consent to partake in the study. Their physical characteristics are shown in Table 1. Exclusion criteria included hypertension, any acute or chronic ophthalmic disorders, and any condition which would render participants incapable of conducting isometric handgrip exercise in either seated (Seated), supine 6°HDT (Supine), or prone 6°HDT (Prone) positions. Except for registration in a database, this study conformed to the Declaration of Helsinki and was approved by the National Medical Ethics Committee (approval no. 0120‐31/2020/9) at the Ministry of Health (Republic of Slovenia).

**TABLE 1:** Participants’ anthropometric characteristics and maximum isometric grip strength obtained in the seated (SEATED), supine 6° head‐down tilt (SUPINE), and prone 6° head‐down tilt (PRONE) positions

			Range	
Variable	Mean	SD	Min	Max
Age (years)	22.5	3.1	19	29
Height (cm)	179.6	5.8	169.0	188.2
Weight (kg)	78.7	12.9	60.2	107.9
Max grip (SEATED; kg)	41.9	6.8	27.2	52.1
Max grip (SUPINE; kg)	43.1	7.1	29.3	53.9
Max grip (PRONE; kg)	42.1	7.1	27.3	51.9

### 2.2 Experimental protocol

**FIGURE 1:**
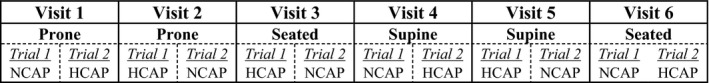
Example of the sequence of the trials for one of the participants. Every participant visited the laboratory on six occasions, with a minimum of 48 h between consecutive visits. During two visits the trials were conducted in a seated position (Seated), during two in the supine 6°HDT position (Supine), and during two in the prone 6°HDT position (Prone). During each visit they completed one trial while breathing normoxic normocapnic air (NCAP) and one trial while breathing normoxic hypercapnic gas mixture (HCAP). The order of the visits, and thus trials, was randomized

**FIGURE 2:**
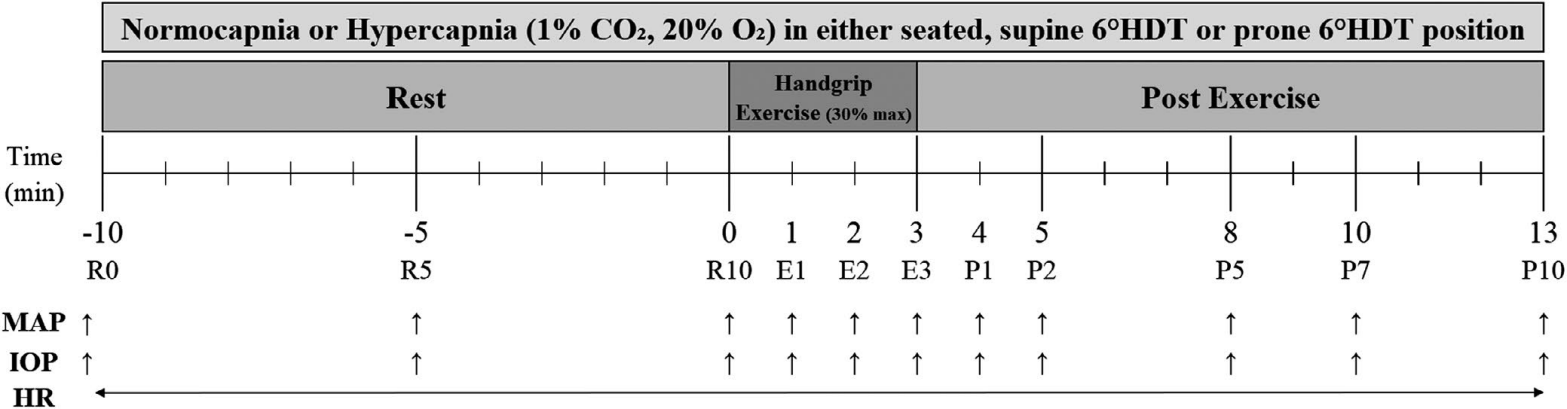
Graphic depiction of the protocol. Each participant underwent two experimental sessions in each of the three positions (seated, supine 6°HDT, and prone 6°HDT), six altogether. During each experimental session, participants conducted two trials; during one trial they breathed normocapnic normoxic air, and during the other normoxic hypercapnic gas mixture (1% CO_2_). (Note: MAP, mean arterial pressure; IOP, intraocular pressure; HR, heart rate)

To assess the effect of the two factors, namely posture (seated, supine 6°HDT, and prone 6°HDT) and breathing mixture (normocapnic and hypercapnic) on IOP during rest and static handgrip exercise, participants were required to visit the laboratory on six occasions, with a minimum of 48 h between consecutive visits. During two visits the trials were conducted in a seated position (Seated), during two in the supine 6°HDT position (Supine), and during two in the prone 6°HDT position (Prone), as seen on Figure 1. The order of the visits, and thus trials, was randomized. Each visit comprised a control trial (Control), in which IOP was measured with the participant in the standard clinical seated position. The Control measurement was followed by two 23‐min trials separated by 30 min. In one 23‐min trial, the participants inspired normoxic normocapnic room air (NCAP) and in the other a normoxic hypercapnic (F_I_CO_2_ = 0.01) gas mixture (HCAP). The NCAP and HCAP trials had an identical protocol, comprising three phases: (i) 10‐min rest, (ii) 3‐min isometric handgrip exercise at 30% of participant's maximum, and (iii) 10‐min recovery (Figure 2). Although we have previously demonstrated no effect of the order of HCAP and NCAP trials on the observed responses (Mekjavic et al., 2020), the order of the trials during the second visit in a given position was reversed. The analysis of the results was conducted on the averaged data of the two trials. To avoid diurnal fluctuations in any of the measured physiological variables, participants were required to visit the laboratory at the same time of the day.

### 2.3 Intraocular pressure measurements in a standard clinical seated position

On arrival at the laboratory, participant's IOP was measured in a standard clinical seated position. This was performed to ensure that participants’ IOP on days they undertook trials in different positions (Control_Seated_, Control_Supine_, and Control_Prone_), did not differ and, therefore, impact the trials’ results. Triplicate measurements were obtained from the right eye using Pulsair IntelliPuff Tonometer (Keeler, Windsor, United Kingdom). The three IOP measurements’ average was taken as a participant's measured Control value in a given visit.

### 2.4 Maximal strength measurement

Maximal handgrip strength in each of the three positions was measured on the days when the participants performed their first trial in a given position. For example, a participant whose schedule is presented in Figure 1, performed a maximal strength measurement in a prone 6°HDT position at the beginning of visit 1, in a seated position at the beginning of visit 3, and in a supine 6°HDT position at the beginning of visit 4.

To measure participants’ maximal isometric handgrip strength, they assumed one of the three positions. For measurement in a seated position, they sat on a stool with their back upright (90°), and for measurements in supine and prone 6°HDT positions, they lay on a bespoke bed. The bed was at a 6° angle and had a padded horseshoe‐shaped headrest attached at its lower part. This allowed the face to protrude from the opening in the headrest while maintaining a relaxed neck and enabled IOP measurement in the prone 6°HDT position. During the maximal strength measurements, participants held the handgrip dynamometer (K‐Force Grip, KINVENT, Montpellier, France) in their right hand with flexion at the elbow maintained at a 90° angle. On instruction, participants conducted maximal isometric handgrip with a handheld dynamometer twice for 5 s with a 60 s rest between the exertions. Strong verbal encouragement was provided throughout both trials. The participant's maximum handgrip strength was determined as the highest force obtained in the two trials.

### 2.5 Normocapnic and hypercapnic exercise trials

Prior to the trials, participants were instrumented for the measurement of impedance electrocardiography (PhysioFlow Q‐Link, Manatec Biomedical, Paris, France), previously validated against the direct Fick method during exercise in healthy participants (Richard et al., 2001; Siebenmann et al., 2015). The PhysioFlow device provided continuous measurement of heart rate (HR, min^−1^).

A blood pressure cuff (Withings model BP‐800, Issy‐les‐Moulineaux, France) was fitted to the participant's left arm before assuming one of the three positions, for measurement of systolic (SAP, mmHg) and diastolic (DAP, mmHg) arterial pressure. Withings BP‐800 sphygmomanometer fulfills the validation criteria of the European Society of Hypertension International Protocol Revision 2010 (Topouchian et al., 2014). Measures of SAP and DAP were used to calculate mean arterial pressure (MAP, mmHg)

(1) $$\mathrm{MAP}=\;\frac{\mathrm{SAP}+2\mathrm{DAP}}{3}$$

Upon instrumentation, participants assumed one of the three positions (Seated, Supine or Prone) and were fitted with a nose clip and mouthpiece connected to a two‐way non‐rebreathing valve (Hans Rudolph Inc., Shawnee, Kansas, USA). During the trial, IOP, SAP, DAP, and HR were recorded toward the end of the 5^th^ and 10^th^ minutes of the rest period (R5 and R10, respectively), each minute of the 3‐min exercise period (E1–E3; isometric handgrip exercise at 30% of their maximum, using a handheld dynamometer) and at the end of the 1^st^, 2^nd^, 5^th^, 7^th^, and 10^th^ minutes of the post‐exercise recovery period (P1, P2, P5, P7, and P10, respectively), as seen in Figure 2. During the 3‐min exercise period, the force exerted by the subjects with the handgrip dynamometer was displayed on a screen (KAPA‐INVENT, 2017), providing them continuous feedback. This was essential, as subjects were instructed to maintain the exerted force at 30% of their previously measured maximal force. To avoid the influence of a Valsalva maneuver on any of the measured variables, all subjects were regularly reminded to keep their neck relaxed and maintain normal respiratory patterns throughout the trial's entirety. Additionally, they were instructed to relax all the muscles not primarily involved in contraction to avoid recruitment of accessory muscles and an increase in blood pressure. At every time point, IOP was measured three times on the right eye, and the average of three measurements was used for the analysis.

The second trial was conducted following a 30‐min rest period. During the rest period, participants were requested to be seated in the upright position. The protocol for both trials was identical, with the exception of the gas inspired. In the NCAP condition, the inspired gas was normocapnic and normoxic room air, whereas in the HCAP condition it was hypercapnic (F_I_CO_2_ = 0.01) and normoxic.

### 2.6 Statistical analyses

All data were assessed for normality using the Shapiro–Wilk and Kolmogorov–Smirnov test of normality. One‐way repeated‐measures ANOVA with a Bonferroni correction was used to investigate any significant difference in participant's IOP when measured in a standard clinical seated position on days when they underwent trials in different positions.

A two‐way repeated‐measures ANOVA was conducted to compare the main effects of all three positions, time, and their interaction effects on all measured variables (IOP, SAP, DAP, MAP, and HR) in each of the two conditions (NCAP and HCAP). When position*time interaction was significant, a one‐way ANOVA with a Bonferroni correction was run as a *post hoc* test. The same *post hoc* test was used to observe the variables’ temporal change in each position (Seated, Supine, or Prone) and each condition (HCAP or NCAP). A paired samples *t*‐test was used to assess if there was any difference between HCAP and NCAP trials in each position.

Descriptive statistics were expressed as mean (SD), and the significance level for all statistical tests in this study was set at *p* < 0.05. Based on the results of a pilot test and previous study (Mekjavic et al., 2020), we determined that for a required observed power of 0.8, minimum eight participants needed to partake in this study. To account for any potential drop‐out, 10 participants were recruited. *A priori* power analysis was conducted using G*Power software (Faul et al., 2007). All other statistical analyses were performed using SPSS (v.25, IBM, NY, USA) software.

## 3 RESULTS

All subjects successfully completed the normocapnic and hypercapnic 3‐min static handgrip exercise performed at 30% of their maximum handgrip strength during the three interventions (Seated, Supine, and Prone). The subjects commented that they could not discern between the two breathing mixtures.

### 3.1 Heart rate (HR, min^−1^)

**FIGURE 3:**
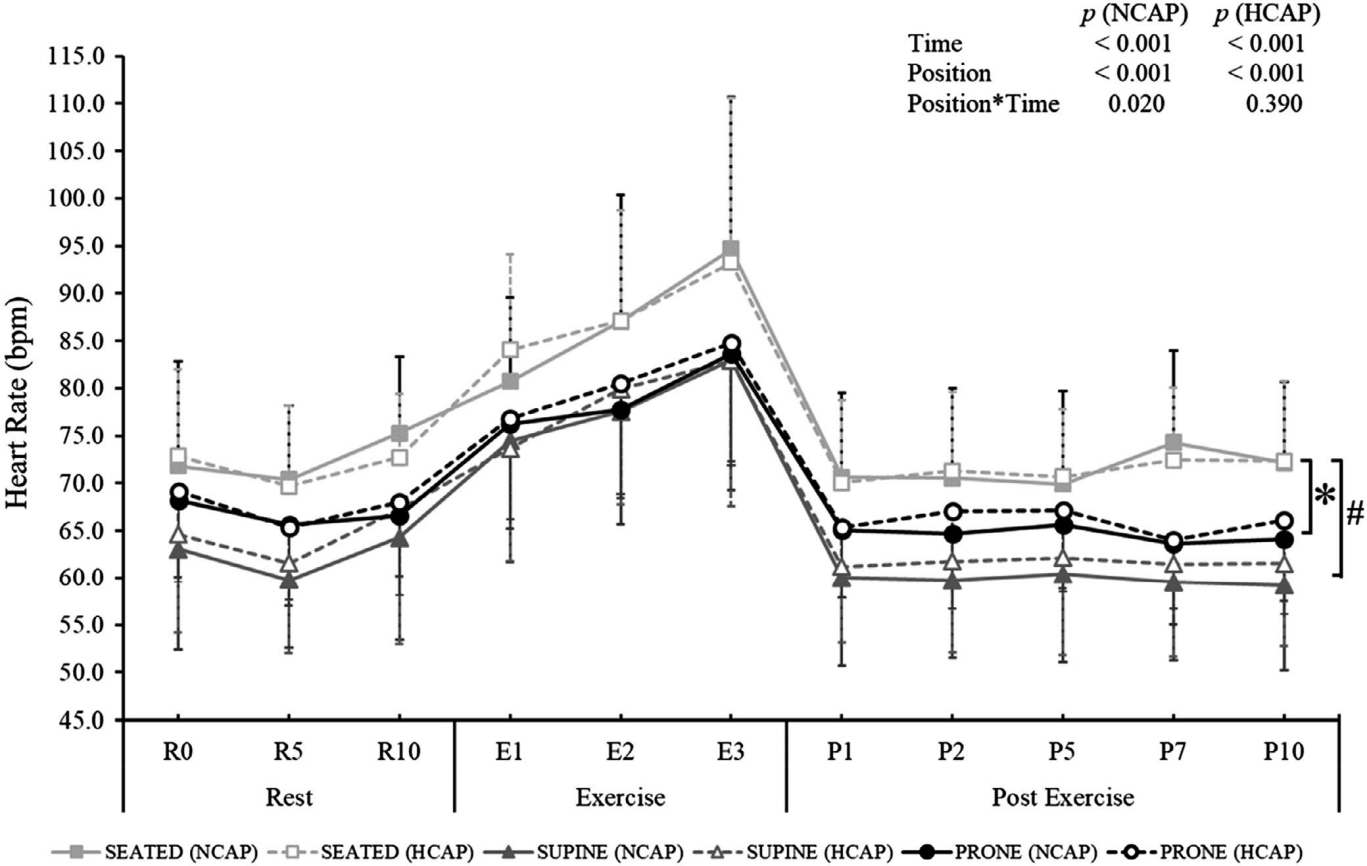
Heart rate (HR) response during normocapnic (NCAP) and hypercapnic (HCAP) trials in seated (SEATED), supine 6°HDT (SUPINE), and prone 6°HDT (PRONE) positions. Each trial consisted of a 10‐min rest period, followed by a 3‐min exercise period (isometric handgrip exercise at 30% participant's maximum) and a 10‐min post‐exercise period. For clarity, results of a two‐way repeated‐measures ANOVA are presented in a table on the graph and the results of a one‐way ANOVA are presented in the text (Note: dashed error bars: HCAP condition; solid error bars: NCAP conditions; * HR during trials in a SEATED position was statistically different to HR during trials in a PRONE position (NCAP and HCAP); # HR during trials in a SEATED position was significantly different to HR during trials in a SUPINE position (NCAP and HCAP); *p* < 0.05)

A significant main effect of position on HR (Figure 3) was observed in both conditions (NCAP: *F*_(2, 18)_ = 22.829, *p *< 0.001; HCAP: *F*_(2, 18)_ = 12.739, *p *< 0.001). *Post hoc* pairwise comparison analysis revealed that in both conditions HR in the Seated trials was significantly higher than in the Supine (NCAP: *p* = 0.001; HCAP *p* = 0.005) and Prone trials (NCAP: *p* = 0.007; HCAP: *p* = 0.044), while HR data in the Supine and Prone trials were statistically indifferent.

A significant main effect of time on HR was observed in all positions (Seated: *F*_(10, 90)_ = 32.644, *p* = 0.002; Supine: *F*_(10, 90)_ = 35.163, *p* < 0.001; Prone: *F*_(10, 90)_ = 50.366, *p* < 0.001). A significant exercise‐induced elevation in HR (E3 compared to R10) was observed in all conditions and positions (0.001 < *p* < 0.008), except during the Seated HCAP trial (*p* = 0.068). A significant decrease in HR immediately upon cessation of exercise (E3 compared to P1) was observed in all positions and conditions (0.001 < *p* < 0.015) and persisted throughout the whole post‐exercise period.

The main effect of the inhaled gas mixture on HR was significant only in the Supine trials (F_(1, 9)_ =10.065, *p* = 0.011). A significant interaction effect of position and time on HR was observed in the NCAP condition (F_(20, 180)_ = 26.085, *p* = 0.020), where HR during the Seated trials was higher compared to HR during the Supine trials at R5, R10, P1, P2, P7, and P10 (0.002 < *p* < 0.035), and higher than during the Prone trials at P7 (*p* = 0.028).

### 3.2 Systolic, diastolic, and mean arterial pressure (SAP, DAP, and MAP, mmHg)

A significant main effect of position on SAP was observed only in the NCAP condition (NCAP: *F*_(2, 18)_ = 7.708, *p* = 0.004). *Post hoc* pairwise comparison revealed that SAP in the Prone trials was significantly lower than in the Seated trials (*p* = 0.018). A main effect of position on DAP was observed in both NCAP and HCAP conditions (NCAP: *F*_(2, 18)_ = 38.852, *p* < 0.001; HCAP: *F*_(2, 18)_ = 45.456, *p* < 0.001) with DAP in the Seated trials being significantly higher than during the Supine (NCAP: *p* < 0.001; HCAP: *p* < 0.001) and Prone (NCAP: *p* < 0.001; HCAP: *p *< 0.001) trials.

Main effect of time on SAP and DAP was observed in all positions (Seated: *F*_(10, 90)_ = 55.279, *p* < 0.001 and *F*_(10, 90)_ = 63.108, *p* < 0.001, respectively; Supine: *F*_(10, 90)_ = 41.150, *p* < 0.001 and *F*_(10, 90)_ = 49.332, *p* < 0.001, respectively; Prone: *F*_(10, 90)_ = 67.933, *p* < 0.001 and *F*_(10, 90)_ = 56.282, *p* < 0.001, respectively), as seen in Table 2. Additionally, a significant effect of position and time interaction on DAP was observed only in HCAP trials (*F*_(20, 180)_ = 1.925, *p* = 0.013), and main effect of inhaled gas mixture on DAP was significant only in the Prone trials (*F*_(1, 9)_ = 16.510, *p* = 0.003), as seen in Table 2.

**TABLE 2:** Average (SD) responses of systolic and diastolic pressure during normoxic hypercapnic (HCAP) and normoxic normocapnic (NCAP) conditions in seated, supine 6°HDT, and prone 6°HDT positions (SEATED, SUPINE, and PRONE, respectively)

Systolic pressure												
		R0	R5	R10	E1	E2	E3	P1	P2	P5	P7	P10
NCAP	SEATED	121 (7)	124 (7)	126 (7)	134 (8)^a^	144 (11)^a^	155 (14)^a^	128 (8)^b^	124 (8)^b^	127 (9)^b^	127 (8)^b^	125 (9)^b^
	SUPINE	126 (10)	123 (9)	122 (7)	132 (9)^a^	139 (12)^a^	149 (10)^a^	129 (10)^b^	123 (8)^b^	119 (20)^b^	124 (7)^b^	123 (8)^b^
	PRONE	124 (8)	120 (6)	120 (7)	126 (8)^a^	133 (8)^a^	142 (12)^a^	124 (8)^b^	122 (8)^b^	121 (6)^b^	120 (7)^b^	121 (8)^b^
HCAP	SEATED	123 (7)	124 (7)	125 (8)	133 (8)^a^	141 (14)	154 (14^)a^	129 (14)^b^	127 (9)^b^	126 (11)^b^	124 (9)^b^	125 (8)^b^
	SUPINE	125 (9)	125 (9)	123 (7)	130 (8)^a^	140 (12)^a^	148 (15)^a^	130 (9)^b^	125 (9)^b^	126 (9)^b^	124 (8)^b^	124 (8)^b^
	PRONE	123 (8)	121 (5)	120 (7)	129 (8)^a^	136 (9)^a^	145 (8)^a^	126 (8)^b^	122 (9)^b^	122 (6)^b^	122 (7)^b^	123 (6)^b^
**Diastolic pressure**												
NCAP	SEATED	77 (6)	77 (8)	77 (10)	83 (11)^a^	92 (9)^a^	100 (7)^a^	74 (7)^b^	75 (10)^b^	76 (9)^b^	76 (7)^b^	78 (7)^b^
	SUPINE	68 (7)	66 (6)	65 (6)	74 (8)^a^	80 (11)^a^	90 (10)^a^	65 (6)^b^	61 (7)^b^	65 (8)^b^	64 (7)^b^	67 (4)^b^
	PRONE	68 (7)	65 (6)	66 (7)	71 (9)	77 (10)^a^	87 (7)^a^	65 (7)^b^	62 (6)^b^	65 (7)^b^	65 (7)^b^	66 (5)^b^
HCAP	SEATED	76 (8)	76 (8)	78 (9)	84 (7)^a^	93 (10)^a^	104 (11)^a^	77 (9)^b^	76 (10)^b^	77 (8)^b^	77 (7)^b^	79 (8)^b^
	SUPINE	67 (6)^c^	69 (6)^c^	66 (6)^c^	72 (8)^c^	81 (11)^c^	89 (9)^a^, ^c^	67 (8)^b^, ^c^	62 (7)^b^, ^c^	67 (5)^b^, ^c^	66 (5)^b^, ^c^	66 (6)^b^, ^c^
	PRONE	69 (6)	67 (4)^c^	70 (6)	73 (7)^c^	79 (8)^a^, ^c^	87 (7)^a^, ^c^	66 (6)^b^, ^c^	65 (6)^b^, ^c^	67 (6)^b^, ^c^	68 (6)^b^, ^c^	66 (6)^b^, ^c^

^a^ Significantly different response during exercise compared to R10.

^b^ Significantly different response during recovery period compared to last minute of exercise (E3).

^c^ Significantly different from SEAT; *p* < 0.05.

**FIGURE 4:**
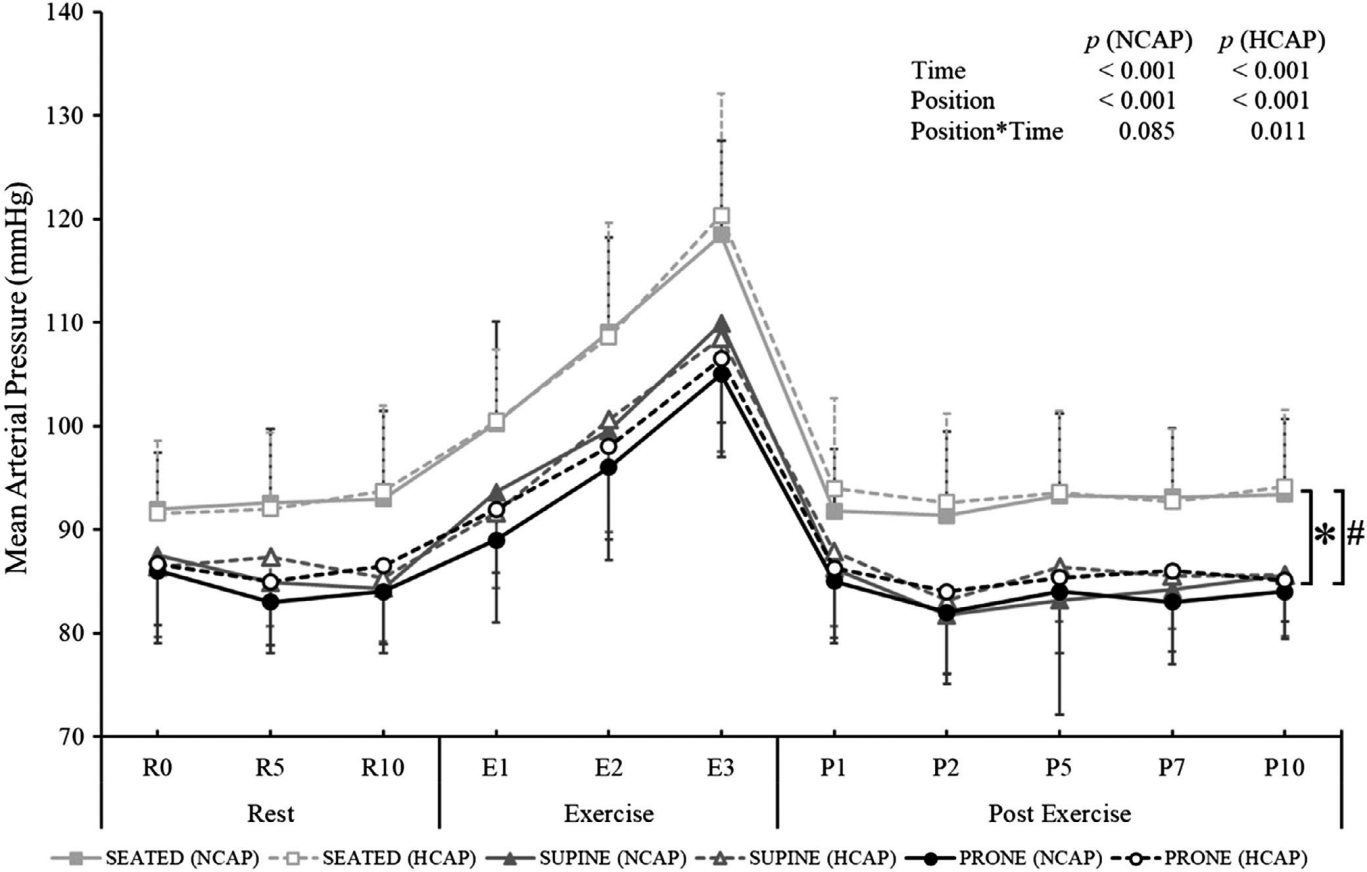
Mean arterial pressure (MAP) response during normocapnic (NCAP) and hypercapnic (HCAP) trials in seated (SEATED), supine 6°HDT (SUPINE), and prone 6°HDT (PRONE) positions. Each trial consisted of a 10‐min rest period, followed by a 3‐min exercise period (isometric handgrip exercise at 30% participant's maximum) and a 10‐min post‐exercise period. For clarity, results of a two‐way repeated‐measures ANOVA are presented on a graph and the results of a one‐way ANOVA are presented in the text (Note: dashed error bars: HCAP condition; solid error bars: NCAP condition; * MAP during trials in a SEATED position was statistically different to MAP during trials in a PRONE position (NCAP and HCAP); # MAP during trials in a SEATED position was significantly different to MAP during trials in a SUPINE position (NCAP and HCAP); *p* < 0.05)

Main effect of position on MAP was observed in both HCAP and NCAP conditions (NCAP: *F*_(2,18)_ = 16.336, *p* < 0.001; HCAP *F*_(2, 18)_ = 24.528, *p* < 0.001), as seen in Figure 4. MAP during the Seated trials was significantly higher compared to the Supine (NCAP: *p* = 0.029; HCAP: *p* = 0.002) and Prone (NCAP: *p* < 0.001; HCAP: *p* = 0.001) trials.

Main effect of time on MAP was present in all positions (Seated: *F*_(10, 90)_ = 75.216, *p* < 0.001; Supine: *F*_(10, 90)_ = 8.890, *p* < 0.001; Prone: *F*_(10, 90)_ = 77.272, *p* < 0.001). MAP increased significantly immediately with exercise (R10 compared to E1) in all positions and conditions (0.001 < *p* < 0.020), except in the Supine HCAP trial (*p* = 0.138). By the 3^rd^ minute of exercise (E3) MAP in all conditions and positions was significantly higher than at R10 (0.001 < *p* < 0.004). Immediately upon cessation of exercise (E3 compared to P1), MAP significantly decreased (*p* < 0.001) and stayed decreased throughout the whole post‐exercise period in all conditions (*p *< 0.001), except in the Supine NCAP trial at P5 (*p* = 1.000).

A significant interaction effect of position and time on MAP was observed only in the HCAP condition (*F*_(80, 180)_ = 18.663, *p* = 0.011). MAP during the Seated trials was significantly higher than during the Supine trials at R10, E1, E3, P2, P5, P7, and P10 (0.012 < *p *< 0.041), and higher than during the Prone trials at R5, E1, E3, P2, P5, P7, and P10 (0.008 < *p *< 0.048).

Main effect of the inhaled gas mixture on MAP was observed only in the Prone trials (*F*_(1, 9)_ = 13.550, *p* = 0.005). *Post hoc* tests revealed this difference was present at R5, R10, T1, T2, P2, and P7 (0.002 < *p* ≤ 0.037).

### 3.3 Intraocular pressure (IOP, mmHg)

As shown in Table 3, no significant difference was found between participants’ IOP measured in a standard clinical seated position on days when they undertook trials in different positions (*p* = 0.189).

**TABLE 3:** Participants’ IOP measured in a standard clinical seated position on days when they undertook trials in different positions

			Range	
Variable	Mean	SD	Min	Max
Control_Seated_	13.6	1.8	9.8	16.6
Control_Supine_	13.8	2.1	10.6	17.3
Control_Prone_	13.2	1.4	10.5	15.9

Note

Control_Seated_ when trials were performed in a seated position, Control_Supine_ when trials were performed in a supine 6°HDT (Supine) position, and Control_Prone_ when trials were performed in a prone 6°HDT (Prone) position.

**FIGURE 5:**
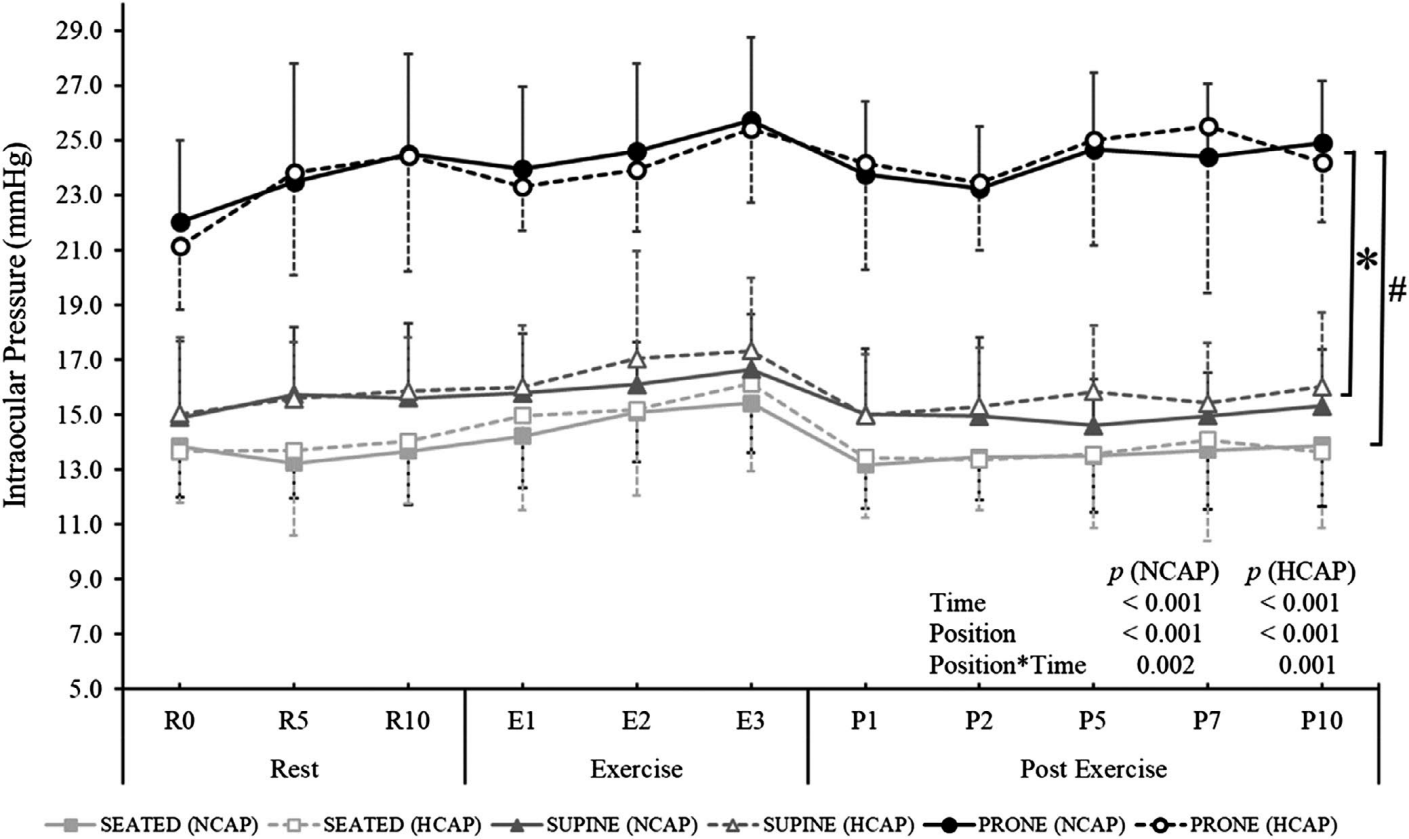
Intraocular pressure (IOP) response during normocapnic (NCAP) and hypercapnic (HCAP) trials in seated (SEATED), supine 6°HDT (SUPINE), and prone 6°HDT (PRONE) positions. Each trial consisted of a 10‐min rest period, followed by a 3‐min exercise period (isometric handgrip exercise at 30% participant's maximum) and a 10‐min post‐exercise period. For clarity, results of a two‐way repeated‐measures ANOVA are presented on a graph and the results of a one‐way ANOVA are presented in the text (Note: dashed error bars: HCAP condition; solid error bars: NCAP conditions; * IOP during trials in a PRONE position was statistically different to IOP during trials in a SUPINE position (NCAP and HCAP); # IOP during trials in a PRONE position was significantly different to IOP during trials in a SEATED position (NCAP and HCAP); *p* < 0.05)

A main effect of position on IOP was present in both NCAP and HCAP conditions (NCAP: *F*_(2, 18)_ = 267.485, *p* < 0.001; HCAP: *F*_(2, 18)_ = 155.436, *p* < 0.001), as seen in Figure 5. In both conditions, IOP in the Prone trials was significantly higher compared to IOP in the Seated (NCAP and HCAP: *p* < 0.001) and Supine (NCAP and HCAP: *p* < 0.001) trials. Additionally, IOP in the Supine trials was higher than in the Seated trials (NCAP: *p* = 0.005; HCAP: *p* = 0.003).

A main effect of time on IOP was observed in all positions (Seated: *F*_(10, 90)_ = 11.914, *p* < 0.001; Supine: *F*_(10, 90)_ = 8.507, *p* < 0.001; Prone: *F*_(10, 90)_ = 22.165, *p* < 0.001), however IOP increased significantly with exercise (R10 compared to E3) only during a HCAP Seated trial (from 14.0 ± 2.3 to 16.1 ± 3.2 mmHg; *p* = 0.042). Furthermore, immediate post‐exercise drop (E3 compared to P1) in IOP was significant only in the Seated and Prone NCAP trials (*p* < 0.048 and *p* < 0.021, respectively).

A significant interaction effect of position and time was observed in both conditions (NCAP: *F*_(20, 180)_ = 2.286, *p* = 0.002; HCAP: *F*_(20, 180)_ = 2.473, *p* = 0.001). *Post hoc* pairwise comparison analysis showed that IOP in Prone trials was higher than IOP in the Seated and Supine trials at all time points (*p* < 0.001) in both (HCAP and NCAP) conditions.

The main effect of the inhaled gas mixture on IOP was significant only in the Supine position (*F*_(1, 9)_ = 10.932, *p* = 0.009). IOP during HCAP trial was significantly higher than during NCAP trial only in the 5^th^ minute of post‐exercise rest (P5; *p* = 0.002).

## 4 DISCUSSION

The main finding of this study is that IOP during rest and static handgrip exercise was significantly greater in the prone 6°HDT position than either the seated or supine 6°HDT positions, confirming our first hypothesis. Additionally, IOP in the prone 6°HDT position was constantly elevated above 21 mmHg. Clinically, long‐term values above 21 mmHg are considered indicative of ocular hypertension (The Royal College of Ophthalmologists, 2016). This was observed in both acute normoxic hypercapnia (HCAP) and normoxic normocapnia (NCAP). In contrast to our previous observations in older participants (Mekjavic et al., 2020), no significant effect of hypercapnia on IOP was observed in any of the positions, in the present population of younger participants. Based on this observation, we could not accept our second hypothesis. IOP measured in a standard seated clinical position (13.5 ± 0.3 mmHg when combined for all three postures) was in line with the normative values for a given population (Martin, 1992; Shiose & Kawase, 1986).

In this study, we did not replicate the length and type of a resistance training as performed on the ISS, since the aim was to observe the general effects of resistive exercise on IOP and not fitness. Additionally, we decided to only look at acute exposures to each condition since prolonged exposure to a prone 6°HDT position could result in too great of a discomfort of the participant.

### 4.1 Posture

It has been suggested that initial and sudden increases in IOP observed during Space missions are most likely due to choroidal engorgement and expansion brought about by headward fluid shifts (Mader et al., 1990, 1993). As reported by Draeger et al. (1993, 1997), IOP increased to 25 mmHg (a 92–114% increase compared to pre‐flight data) within 16 min upon reaching Earth's orbit. Elevations in IOP were also observed during parabolic flights (Anderson et al., 2016; Frey et al., 1993) and bed rest in both the horizontal and 6°HDT supine positions (Anderson et al., 2016, 2017; Carlson et al., 1987; Eklund et al., 2016; Lam & Douthwaite, 1997; Laurie et al., 2017). The higher IOP values observed in a horizontal prone position (Anderson et al., 2016, 2017; Lam & Douthwaite, 1997), are exacerbated by 6°HDT (Mekjavic et al., 2020), which concurs with the present findings. Additionally, Anderson et al. (2016) showed with a series of measurements conducted in the horizontal supine and prone positions, and during parabolic flights, that IOP is not governed only by fluid shifts, but also by changes in hydrostatic gradients produced by changes in the direction of the gravity vector. During parabolic flights, Anderson et al. (2016) observed IOP values (16.3 ± 2.7 mmHg) that were between the values measured on the ground in the horizontal supine (13.7 ± 3.0 mmHg) and prone (20.3 ± 2.6 mmHg) positions. IOP values obtained by Anderson et al. (2016) in all three conditions were remarkably lower than IOP values measured during spaceflight (Draeger et al., 1993, 1997). This could be attributed to the fact that measurements in the supine and prone position were conducted in a horizontal and not 6°HDT position, and insufficient time was allocated for the IOP to stabilize in each position. Although in this study IOP in supine 6°HDT increased to 15.7 ± 2.3 mmHg (NCAP and HCAP combined) by the end of the initial 10‐min rest and was not significantly different from the seated values (13.8 ± 2.1 mmHg for NCAP and HCAP combined), IOP in the prone 6°HDT position increased to 24.5 ± 3.9 mmHg (NCAP and HCAP combined), values that are very similar to those reported by Draeger et al. (1993, 1997). It can be, therefore, speculated that fluid shifts and the direction of the gravity vector in prone 6°HDT better mimic the effects of microgravity, hereby making it a more appropriate ground‐based simulation model of acute ocular changes that occur in microgravity, than supine 6°HDT.

### 4.2 Resistive exercise

A multitude of exercise countermeasures have proven to be only partially effective in preventing the adaptation of physiological systems to microgravity. In an effort to mitigate musculoskeletal atrophy, astronauts on the ISS conduct daily exercise, using equipment specially designed for microgravity. Their daily training on average lasts 2 h and consists of a combination of aerobic and resistive exercises using the Advanced Resistive Exercise Device (ARED) (Marshall‐Bowman et al., 2013).

Static exercise invokes marked increases in MAP (Avunduk et al., 1999; Lind, 1970), which are further exacerbated by a Valsalva maneuver (Narloch & Brandstater, 1995; Zebrowska et al., 2013). Narloch and Brandstater (1995) reported that during a five repetition maximum (RM) leg press, MAP increased 110 mmHg more (183 vs. 293 mmHg) when Valsalva maneuver was performed compared to slow exhalation during concentric contraction. In this study, MAP increased significantly with exercise in all positions and conditions. Prior to testing, all the participants were told to avoid holding their breath, but since the breathing parameters were only observed visually and not monitored, we cannot ascertain whether all the participants successfully adhered to the instructions. Similarly, even though astronauts are trained to execute all exercise actions appropriately, it is likely that Valsalva manoeuvres are still occasionally performed during a strength training exercise on the ARED onboard the ISS.

Reports regarding the behavior of IOP during static exercise are contradictory. The majority of studies reported significant increases of IOP with exercise (Bakke et al., 2009; Dickerman et al., 1999; Mekjavic et al., 2020; Vieira et al., 2006), while others reported decreases (Lanigan et al., 1989), or even no change (Marcus et al., 1974; Robinson et al., 1986), as observed in this study. When IOP did increase with static exercise, this increase was observed both in the presence (Dickerman et al., 1999; Vieira et al., 2006) and absence (Bakke et al., 2009; Mekjavic et al., 2020; Vieira et al., 2006) of a Valsalva maneuver. Studies investigating the relationship between MAP and IOP, also report contradictory results. Some studies found that IOP increases parallel with MAP during static exercise (Bakke et al., 2009; Mekjavic et al., 2020), whereas others reported a decrease (Lanigan et al., 1989) or found no change in IOP despite the increase in MAP (Marcus et al., 1974; Robinson et al., 1986). In this study, MAP increased significantly in all positions and conditions; however, significant IOP elevations were only observed in the Seated position during hypercapnic exercise.

A drop in IOP has been observed following exercise, after short or prolonged bed rest and upon return to Earth. Whereas post‐exercise drop in IOP, as in our study, has been attributed to a decrease in plasma pH, and an increase in plasma osmolality and lactate (Marcus et al., 1970), the decrement in IOP following exposure to simulated and actual microgravity, has been hypothesized to result from a sudden drop in choroidal volume, consequently resulting in a decreased aqueous volume and lower than expected IOP (Mader, 1991).

It has recently been reported (Fischman et al., 2018) that low‐level resistance exercise does not affect ICP in either the seated or supine position. Even though IOP in this study did not increase with exercise in any of the positions while in normocapnia, IOP values obtained in a Prone trial suggest that the prone 6°HDT rather than the supine 6°HDT position might be a better ground‐based simulation of the effects of acute microgravity on IOP, therefore a similar analysis should be performed to investigate the ICP responses in a prone position.

### 4.3 Hypercapnia

The headaches often reported by, otherwise, healthy astronauts (Law et al., 2014) onboard the ISS have been attributed to the elevated levels of ambient CO_2_. Consequently, it was speculated that this ambient hypercapnia‐induced cerebral vasodilation in conjunction with decreased venous drainage that occurs due to the loss of hydrostatic fluid gradient, might be a contributing factor toward the development of ocular changes observed in astronauts (Marshall‐Bowman et al., 2013; Marshall‐Goebel, Mulder, Donoviel, et al., 2017). CO_2_ is a potent vasodilator that has been shown to cause increases in IOP (Laurie et al., 2017; Mekjavic et al., 2020) and MAP (Sechzer et al., 1960). In this study, difference in the IOP response to hypercapnia and normocapnia during isometric handgrip exercise was observed only in a Seated position, where IOP increased significantly in a HCAP trial (from 14.0 ± 2.3 at R10 to 16.1 ± 3.2 mmHg at T3; *p* = 0.042) but not in a NCAP trial (from 13.7 ± 2.4 at R10 to 15.4 ± 2.2 mmHg at T3; *p* = 0.303). No such changes were observed in any other position, suggesting that IOP increases caused by cephalad fluid displacement in supine and prone 6°HDT positions potentially negated any changes that would have occurred due to hypercapnia.

Jaki Mekjavic et al. (2016) conducted examinations of the retina using optical coherence tomography (OCT) before and after 10‐day bed rest, with subjects inspiring either a hypoxic or a hypercapnic breathing mixture identical to this study (F_I_CO_2_ = 0.01). Whereas a vasoactive effect of both hypercapnia and hypoxia was observed on the vessels in the neuroretina, the vessels in the choroid were predominantly affected by the hydrostatic component. In part, this may explain the observation in this study where the highest IOP levels were recorded in the prone 6°HDT position.

Previously, we showed that in older males (range: 48–65 years) the IOP responses to static handgrip exercise in prone 6°HDT position are significantly elevated in hypercapnic compared to normocapnic conditions (Mekjavic et al., 2020). It is important to mention that participants in both of the studies that observed elevations in IOP when exposed to acute hypercapnia were older (Laurie et al. (2017): 25–49 years; Mekjavic et al. (2020): 48–65 years) than the participants included in this study (range: 19–29 years). Therefore, the discrepancy in the results could also be attributed to the diminished ventilatory response to hypercapnia in elderly (Kronenberg & Drage, 1973; Peterson et al., 1981). Interestingly, Kronenberg and Drage (1973) observed a 40% lower hypercapnic ventilatory drive in elderly (64–73 years old) compared to younger (22–30 years old) men.

### 4.4 Limitations

During the pilot phase of this study, we tested three portable tonometers (Pulsair IntelliPuff; iCare IC200, Icare Finland Oy, Finland; Tono‐Pen, Reichert Technologies, USA) for the measurement of IOP. Due to the nature of the experimental arrangement, Pulsair IntelliPuff was used in the study to avoid the use of an eye anesthetic and to make it possible to measure IOP in all three positions. Pulsair IntelliPuff tonometer has been reported to overestimate measured IOP values compared to those measured with Goldmann applanation tonometry. Furthermore, an excellent agreement (intraclass correlation coefficient = 0.75) between the data collected with Pulsair IntelliPuff and Goldmann applanation tonometer showed that Pulsair IntelliPuff is an appropriate tool for measuring IOP in normo‐ and hypertensive individuals (Hubanova et al., 2015).

A limitation of this study is the lack of control resting trials in all three positions and both conditions during which participants would rest for the entire 23 minutes, assuming the postures tested. The 10‐min rest prior to the exercise in this study, might not have been of sufficient duration to allow IOP to stabilize, especially in the prone 6°HDT position. A resting trial would allow us to observe any drift in the measured variables. If such data were available, and indicated a significant drift, then the presented IOP data could have been baseline corrected to reveal the true physiological responses of the variables.

Lastly, this study only included younger male participants to avoid the presence of any age‐related (ocular) illnesses. Previously, we showed that in older males IOP in prone 6°HDT increases with exercise and is further exacerbated by hypercapnia (Mekjavic et al., 2020). Data shows that female astronauts are less likely to develop SANS (Mader et al., 2011; Mark et al., 2014), potentially indicating that certain physiological differences between the sexes influence the manifestation of ocular changes observed in Space. Therefore, to assess the influence of posture on IOP without the interference of these potential differences, we decided to include only younger male participants in this study.

## 5 CONCLUSION

This study demonstrates that posture (i.e., seated, 6°HDT supine, and 6°HDT prone) significantly affects IOP, MAP, and HR during rest and 3‐min static handgrip exercise. Whereas mild hypercapnia was previously reported to exacerbate IOP elevations in older males (Mekjavic et al., 2020), no such responses were observed in the population of younger males participating in this study. The prone 6°HDT position would appear to be more suitable for simulation of ocular changes that occur in acute microgravity than supine 6°HDT.

## CONFLICT OF INTEREST

The authors declare that they have no conflict of interest.

## AUTHORS’ CONTRIBUTIONS

The experiments were performed at the Jozef Stefan Institute (Ljubljana, Slovenia). PJM and IBM conceived the study. TM, PJM, and IBM designed the study. All authors contributed to the data acquisition. TM analyzed the data. TM prepared the figures and tables. TM drafted the manuscript. All authors edited and revised the manuscript. All authors read and approved the final version of the manuscript.

## CONSENT TO PARTICIPATE

Written informed consent was obtained from all individual participants included in the study.

## CONSENT FOR PUBLICATION

All authors read the final version of the manuscript and approved its publication.

## CODE AVAILABILITY

Not applicable.
